# Supplementary material for: Prevalence and associated factors of dyslipidemia among adults with coexisting chronic disease in Ethiopia: A systematic review and meta-analysis
Source: PLoS One. 2025 Apr 29;20(4):e0320119. doi: 10.1371/journal.pone.0320119 (PMC12040176; doi:10.1371/journal.pone.0320119)
Supplement: S2 Table — (DOCX) [file pone.0320119.s002.docx]

Pubmed database search: results 108

| # | Concept | Searches | Results |
| --- | --- | --- | --- |
| 1 | Dyslipidemia | ("Dyslipidemias"[Mesh]) OR "Hypercholesterolemia"[Mesh] OR dyslipid*[tw] OR dyslipidemia[tw] OR “dyslipidaemia”[tw] OR “lipid profile”[tw] OR “Mets”[tw] OR “ metabolic syndrome* ”[tw] OR “ Hypercholesterol* ”[tw] OR “hyperlipidemia” OR “lipid disorder” | 234,791 |
| 2 | Prevalence | "Prevalence"[Mesh] OR Prevalence [tw] OR Burden[tw] OR Magnitude [tw] | 1,454,512 |
| 3 | Associated factors | "Precipitating Factors"[Mesh] OR "Risk Factors"[Mesh] OR “Associated factors”[tw] OR “risk factors”[tw] OR “Factors affecting”[tw] OR “Predictor” OR “Determinant” | 1,643,154 |
| 4 | Ethiopia | "Ethiopia" [Mesh] OR Ethiopia [tw] | 31,052 |
| 5 | #1 AND #2 AND #3 AND #4 | (((("Dyslipidemias"[Mesh]) OR "Hypercholesterolemia"[Mesh] OR dyslipid*[tw] OR dyslipidemia[tw] OR "dyslipidaemia"[tw] OR "lipid profile"[tw] OR "Mets"[tw] OR " metabolic syndrome* "[tw] OR " Hypercholesterol* "[tw] OR "hyperlipidemia" OR "lipid disorder") AND ("Prevalence"[Mesh] OR Prevalence [tw] OR Burden[tw] OR Magnitude [tw])) AND ("Precipitating Factors"[Mesh] OR "Risk Factors"[Mesh] OR "Associated factors"[tw] OR "risk factors"[tw] OR "Factors affecting"[tw] OR "Predictor" OR "Determinant")) AND ("Ethiopia" [Mesh] OR Ethiopia [tw]) | 108 |

Embase database search: 247 results

| # | Concept | Searches | Results |
| --- | --- | --- | --- |
| 1 | Dyslipidemia | (('dyslipidemia'/exp OR dyslipidemia OR 'metabolic syndrome x'/exp OR 'metabolic syndrome x' OR 'hypercholesterolemia'/exp OR hypercholesterolemia OR 'hyperlipidemia'/exp OR hyperlipidemia)) | 396,499 |
| 2 | Prevalence/ Associated factors | ('prevalence'/exp OR prevalence) OR ('associated factor'/exp OR 'associated factor') | 1,428,518 |
| 3 | Ethiopia | (''associated factor '/exp OR ethiopia) | 43,746 |
| 4 | #1 AND #2 AND #3 | (('dyslipidemia'/exp OR dyslipidemia OR 'metabolic syndrome x'/exp OR 'metabolic syndrome x' OR 'hypercholesterolemia'/exp OR hypercholesterolemia OR 'hyperlipidemia'/exp OR hyperlipidemia) AND ('prevalence'/exp OR prevalence) OR 'associated factor'/exp OR 'associated factor') AND ('ethiopia'/exp OR ethiopia) | 247 |
