# Supplementary material for: Prevalence and associated factors of dyslipidemia among adults with coexisting chronic disease in Ethiopia: A systematic review and meta-analysis
Source: PLoS One. 2025 Apr 29;20(4):e0320119. doi: 10.1371/journal.pone.0320119 (PMC12040176; doi:10.1371/journal.pone.0320119)
Supplement: S3 Table — (PDF) [file pone.0320119.s003.pdf]

JBI CRITICAL APPRAISAL CHECKLIST FOR STUDIES REPORTING PREVALENCE DATA																							
		**1. Was the sample frame appropriate to address the target population?**		**2. Were study participants recruited in an appropriate way?**		**3. Was the sample size adequate?**		**4. Were the study subjects and setting described in detail?**		**5. Was data analysis conducted with sufficient coverage of the identified sample?**		**6. Were valid methods used for the identification of the condition?**		**7. Was the condition measured in a standard, reliable way for all participants?**		**8. Was there appropriate statistical analysis?**		**9. Was the response rate adequate, and if not, was the low response rate managed appropriately?**		**Total score**			
**S.N**	**Study ID**	Reviewer1	Reviewer2	Reviewer1	Reviewer2	Reviewer1	Reviewer2	Reviewer1	Reviewer2	Reviewer1	Reviewer2	Reviewer1	Reviewer2	Reviewer1	Reviewer2	Reviewer1	Reviewer	Reviewer1	Reviewer2	**Reviewer1**	**Reviewer2**	**Average score**	**%**
1	Addisu et al. 2023	1	1	0	0	1	0	1	1	1	1	1	1	1	1	1	1	1	1	**8**	**7**	**7.5**	**83**
2	Assefa et al. 2023	1	1	1	1	0	1	1	1	1	1	1	1	1	1	1	1	1	1	**8**	**9**	**8.5**	**94**
3	Kelem et al. 2023	1	1	1	1	1	1	1	1	1	1	1	1	1	1	1	1	1	1	**9**	**9**	**9**	**100**
4	Mohammed et al. 2023	1	1	0	0	1	1	1	1	1	1	1	1	1	1	1	1	1	1	**8**	**8**	**8**	**89**
5	Abdissa and Hirpa 2022	1	1	1	1	1	1	1	1	1	1	1	1	1	1	1	1	1	1	**9**	**9**	**9**	**100**
6	Gebreyesus et al. 2022	1	1	1	1	1	1	1	1	1	1	1	1	1	1	1	1	1	1	**9**	**9**	**9**	**100**
7	Hashim et al. 2022	1	1	1	0	1	1	1	1	1	1	1	1	1	1	1	1	1	1	**9**	**8**	**8.5**	**94**
8	Kabtu and Tsegaw 2022	1	1	0	1	1	0	1	0	0	0	1	1	1	1	1	1	1	1	**7**	**6**	**6.5**	**72**
9	Letta et al. 2022	1	1	1	1	1	1	1	1	1	1	1	1	1	1	1	1	1	1	**9**	**9**	**9**	**100**
10	Lissane et al. 2022	1	1	1	1	0	1	1	1	0	1	1	1	1	1	1	1	0	0	**6**	**8**	**7**	**78**
11	Sahiledengle et al. 2022	1	1	1	1	1	1	1	1	1	1	1	1	1	1	0	1	1	1	**8**	**9**	**8.5**	**94**
12	Timerga et al. 2022	1	1	1	0	1	1	1	1	1	1	1	1	1	1	1	1	1	1	**9**	**8**	**8.5**	**94**
13	Woldeyes et al. 2022	1	1	1	1	1	1	1	1	1	1	1	1	1	1	0	1	1	1	**8**	**9**	**8.5**	**94**
14	Woldeyes et al. 2022	1	1	1	1	1	1	1	1	1	1	1	1	1	1	1	0	1	1	**9**	**8**	**8.5**	**94**
15	Amogne et al. 2021	1	1	0	1	0	1	1	1	1	1	1	0	1	1	1	1	1	1	**7**	**8**	**7.5**	**83**
16	Bogale and Aderaw 2021	1	1	1	1	1	0	1	1	1	1	1	1	0	1	1	1	1	1	**8**	**8**	**8**	**89**
17	Challa et al. 2021	1	1	0	1	1	1	1	1	1	1	1	1	1	1	1	1	1	1	**8**	**9**	**8.5**	**94**
18	Fiseha et al. 2021	1	1	0	0	1	1	1	1	1	1	1	1	1	1	1	1	1	1	**8**	**8**	**8**	**89**
19	Haile et al. 2021	1	1	1	0	1	1	1	1	1	1	1	1	1	1	1	1	1	1	**9**	**8**	**8.5**	**94**
20	Hirigo et al. 2021	1	1	1	1	1	0	1	1	1	1	1	1	1	1	1	1	1	1	**9**	**8**	**8.5**	**94**
21	Kifl et al. 2021	1	1	1	1	1	1	1	1	1	1	1	1	0	1	1	1	1	1	**8**	**9**	**8.5**	**94**
22	Worku et al. 2021	1	1	1	1	1	1	1	1	1	1	1	1	1	1	1	1	1	1	**9**	**9**	**9**	**100**
23	Abdu et al. 2020	1	1	0	0	0	0	1	1	1	1	1	1	1	1	1	1	1	1	**7**	**7**	**7**	**78**
24	Duguma et al. 2020	1	1	1	0	1	0	1	1	1	1	1	1	1	1	1	1	1	1	**9**	**7**	**8**	**89**
25	Haile and Timerga 2020	1	1	0	1	1	1	1	1	1	1	1	1	1	1	1	1	1	1	**8**	**9**	**8.5**	**94**
26	Kemal et al. 2020	1	1	1	1	1	1	1	1	1	1	1	1	1	1	1	1	1	1	**9**	**9**	**9**	**100**
27	Teshome et al. 2020	1	1	1	1	1	1	1	1	1	1	1	1	1	1	1	0	1	1	**9**	**8**	**8.5**	**94**
28	Timerga et al. 2020	1	0	1	1	1	1	1	1	1	1	1	1	1	1	1	1	1	1	**9**	**8**	**8.5**	**94**
29	Zerga and Bezabih 2020	1	1	1	1	1	1	1	1	1	1	1	1	1	1	1	1	1	1	**9**	**9**	**9**	**100**
30	Zewudie et al. 2020	1	1	0	0	1	1	1	1	1	1	1	1	1	1	1	1	1	1	**8**	**8**	**8**	**89**
31	Bahrey et al. 2019	1	1	1	1	0	0	1	1	1	1	1	1	1	1	1	1	1	1	**8**	**8**	**8**	**89**
32	Gebre and Assefa 2019	1	1	1	1	1	1	1	1	1	1	1	1	0	0	1	1	1	1	**8**	**8**	**8**	**89**
33	Gebremeskel et al. 2019	1	1	1	1	1	1	1	1	1	1	1	1	0	1	1	1	1	1	**8**	**9**	**8.5**	**94**
34	Wube et al. 2019	1	1	1	1	1	1	1	1	1	1	1	1	1	1	1	1	1	1	**9**	**9**	**9**	**100**
35	Adal et al. 2018	1	1	0	1	0	0	1	1	1	1	1	1	1	1	1	1	1	1	**7**	**8**	**7.5**	**83**
36	Asaye et al. 2018	1	1	1	1	1	1	1	1	1	1	1	1	1	1	1	1	1	1	**9**	**9**	**9**	**100**
37	Ataro et al. 2018	1	1	1	1	1	1	1	1	1	1	1	1	1	1	1	1	1	1	**9**	**9**	**9**	**100**
38	Belete et al. 2018	1	1	1	1	1	0	1	1	1	1	1	1	1	1	1	1	1	1	**9**	**8**	**8.5**	**94**
39	Birarra and Gelayee 2018	1	1	1	1	1	1	1	1	1	1	0	0	0	0	1	1	1	1	**7**	**7**	**7**	**78**
40	Bosho et al. 2018	1	1	1	1	1	1	1	1	1	1	1	1	1	1	1	1	1	1	**9**	**9**	**9**	**100**
41	Gebrie et al. 2018	1	1	1	1	0	0	1	1	1	1	1	1	1	1	1	1	1	1	**8**	**8**	**8**	**89**
42	Hirigo and Geleta 2018	1	1	1	1	1	1	1	1	1	1	1	1	1	1	1	1	1	1	**9**	**9**	**9**	**100**
43	Bekele et al. 2017	1	1	1	1	0	0	1	1	1	0	1	1	1	1	1	1	1	1	**8**	**7**	**7.5**	**83**
44	Tadewos et al. 2017	1	1	1	1	1	1	1	1	1	1	1	1	0	0	1	1	1	1	**8**	**8**	**8**	**89**
45	Woyesa et al. 2017	1	1	1	1	1	1	1	1	1	1	1	1	1	1	1	1	1	1	**9**	**9**	**9**	**100**
46	Abebe et al. 2016	1	1	1	1	1	1	1	1	1	1	1	1	1	0	1	1	1	1	**9**	**8**	**8.5**	**94**
47	Hirigo and Tesfaye 2016	1	1	0	0	0	1	1	1	1	1	1	1	1	1	1	1	1	1	**7**	**8**	**7.5**	**83**
48	Ambachew et al. 2015	1	1	0	0	0	0	1	1	1	1	1	1	1	1	1	1	1	1	**7**	**7**	**7**	**78**
49	Mohammed et al. 2015	1	1	1	1	1	1	1	1	1	1	1	1	1	1	1	1	1	1	**9**	**9**	**9**	**100**
50	Tachebele et al. 2014	1	1	1	1	1	1	1	1	1	1	1	1	1	1	1	1	1	1	**9**	**9**	**9**	**100**
51	Tesfaye et al. 2014	1	1	1	1	1	0	1	1	1	1	1	1	1	1	0	1	1	1	**8**	**8**	**8**	**89**

CHECKLIST FOR ANALYTICAL CROSS SECTIONAL STUDIES																				
		**1. Were the criteria for inclusion in the sample clearly defined?**		**2. Were the study subjects and the setting described in detail?**		**3. Was the exposure measured in a valid and reliable way?**		**4. Were objective, standard criteria used for measurement of the condition?**		**5. Were confounding factors identified?**		**6. Were strategies to deal with confounding factors stated?**		**7. Were the outcomes measured in a valid and reliable way?**		**8. Was appropriate statistical analysis used?**		**Total score**			
**S.N**	**Study ID**	Reviewer1	Reviewer2	Reviewer1	Reviewer2	Reviewer1	Reviewer2	Reviewer1	Reviewer2	Reviewer1	Reviewer2	Reviewer1	Reviewer2	Reviewer 1	Reviewer 2	Reviewer 1	Reviewer 2	**Reviewer1**	**Reviewer2**	**Average score**	**%**
1	Fentie and Yibabie, 2023	1	1	1	1	1	1	1	1	1	1	1	0	1	1	1	1	**8**	**7**	**7.5**	**94**
2	Kassaw et al. 2022	1	1	1	1	1	1	1	1	1	1	1	0	1	1	1	1	**8**	**7**	**7.5**	**94**
3	Nigatie et al. 2022	1	1	1	1	1	1	1	1	0	0	0	0	1	1	1	1	**6**	**6**	**6**	**75**
4	Woldu et al. 2022	1	1	1	1	1	1	1	1	0	0	0	0	1	1	1	1	**6**	**6**	**6**	**75**
5	Woyesa et al. 2021	1	1	1	1	1	1	1	1	0	0	0	0	1	1	1	1	**6**	**6**	**6**	**75**
6	Kumie et al. 2020	1	1	1	1	1	1	1	1	1	1	0	0	1	1	1	1	**7**	**7**	**7**	**88**
7	Bune et al. 2019	1	1	1	1	1	1	1	1	1	1	1	1	1	1	1	1	**8**	**8**	**8**	**100**
8	Abebe et al. 2014	1	1	1	1	1	1	1	1	1	1	0	0	1	1	1	1	**7**	**7**	**7**	**88**

**CHECKLIST FOR COHORT STUDIES**																											
		**1. Were the two groups similar and recruited from the same population?**		**2. Were the exposures measured similarly to assign people to both exposed and unexposed groups?**		**3. Was the exposure measured in a valid and reliable way?**		**4. Were confounding factors identified?**		**5. Were strategies to deal with confounding factors stated?**		**6. Were the groups/participants free of the outcome at the start of the study (or at the moment of exposure)?**		**7. Were the outcomes measured in a valid and reliable way?**		**8. Was the follow up time reported and sufficient to be long enough for outcomes to occur?**		**9. Was follow up complete, and if not, were the reasons to loss to follow up described and explored?**		**10. Were strategies to address incomplete follow up utilized?**		**11. Was appropriate statistical analysis used?**		**Total score**			
**S.N**	**Study ID**	Reviewer1	Reviewer2	Reviewer1	Reviewer2	Reviewer1	Reviewer2	Reviewer1	Reviewer2	Reviewer1	Reviewer2	Reviewer1	Reviewer2	Reviewer 1	Reviewer 2	Reviewer 1	Reviewer 2	Reviewer 1	Reviewer 2	Reviewer 1	Reviewer 2	Reviewer 1	Reviewer 2	**Reviewer1**	**Reviewer2**	**Average score**	**%**
1	Fanta et al. 2021	1	1	1	1	1	1	1	1	1	1	1	1	1	1	1	1	1	1	1	1	1	1	**11**	**11**	**11**	**100**
2	Yazie 2020	1	1	1	1	1	1	1	1	1	1	1	1	1	1	1	1	1	1	1	1	1	1	**11**	**11**	**11**	**100**

CHECKLIST FOR CASE CONTROL STUDIES																									
		1. Were the groups comparable other than the presence of disease in cases or the absence of disease in controls?		2. Were cases and controls matched appropriately?		3. Were the same criteria used for identification of cases and controls?		4. Was exposure measured in a standard, valid and reliable way?		5. Was exposure measured in the same way for cases and controls?		6. Were confounding factors identified?		7. Were strategies to deal with confounding factors stated?		8. Were outcomes assessed in a standard, valid and reliable way for cases and controls?		9. Was the exposure period of interest long enough to be meaningful?		10. Was appropriate statistical analysis used?		Total score			
**S.N**	**Study ID**	Reviewer1	Reviewer2	Reviewer1	Reviewer2	Reviewer1	Reviewer2	Reviewer1	Reviewer2	Reviewer1	Reviewer2	Reviewer1	Reviewer2	Reviewer 1	Reviewer 2	Reviewer 1	Reviewer 2	Reviewer 1	Reviewer 2	Reviewer 1	Reviewer 2	**Reviewer1**	**Reviewer2**	**Average score**	**%**
1	Gebremedhin et al. 2021	1	1	0	0	1	1	1	1	1	1	1	1	1	1	1	1	1	1	1	1	**9**	**9**	**9**	**90**
