# Supplementary figures and images for: Prevalence and associated factors of dyslipidemia among adults with coexisting chronic disease in Ethiopia: A systematic review and meta-analysis
Source: PLoS One. 2025 Apr 29;20(4):e0320119. doi: 10.1371/journal.pone.0320119 (PMC12040176; doi:10.1371/journal.pone.0320119)

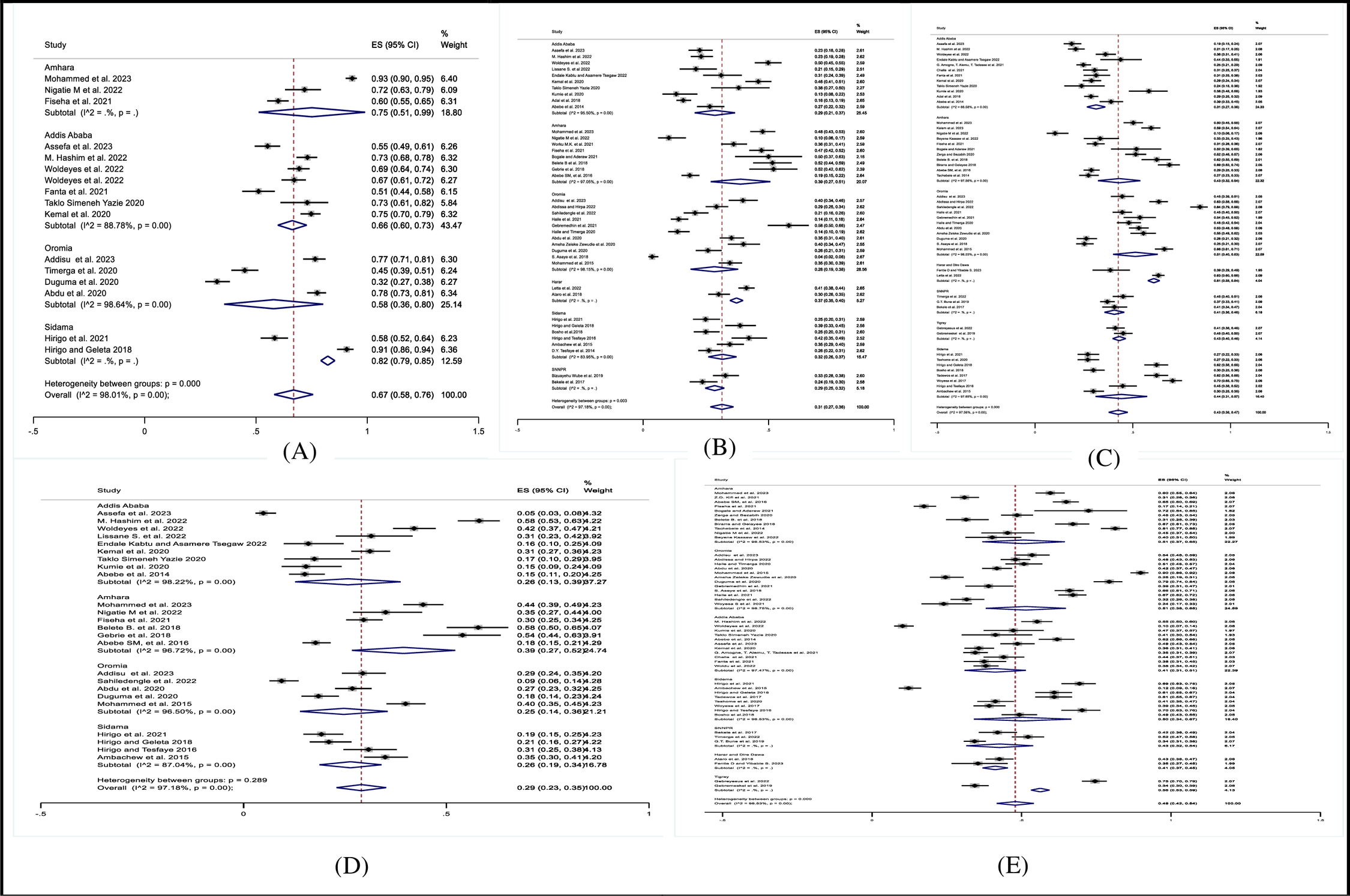

Supplement: S1 Fig — Forest plots of pooled prevalence of overall dyslipidemia and dyslipidemia components among patients in Ethiopia by region. A, Forest plot displaying the pooled prevalence of overall dyslipidemia by region. B, Forest plot displaying the pooled prevalence of TC by region. C, Forest plot displaying the pooled prevalence of TG by region. D, Forest plot displaying the pooled prevalence of LDL-c by region. E, Forest plot displaying the pooled prevalence of HDL-c by region. (TIF) [file pone.0320119.s004.tif]

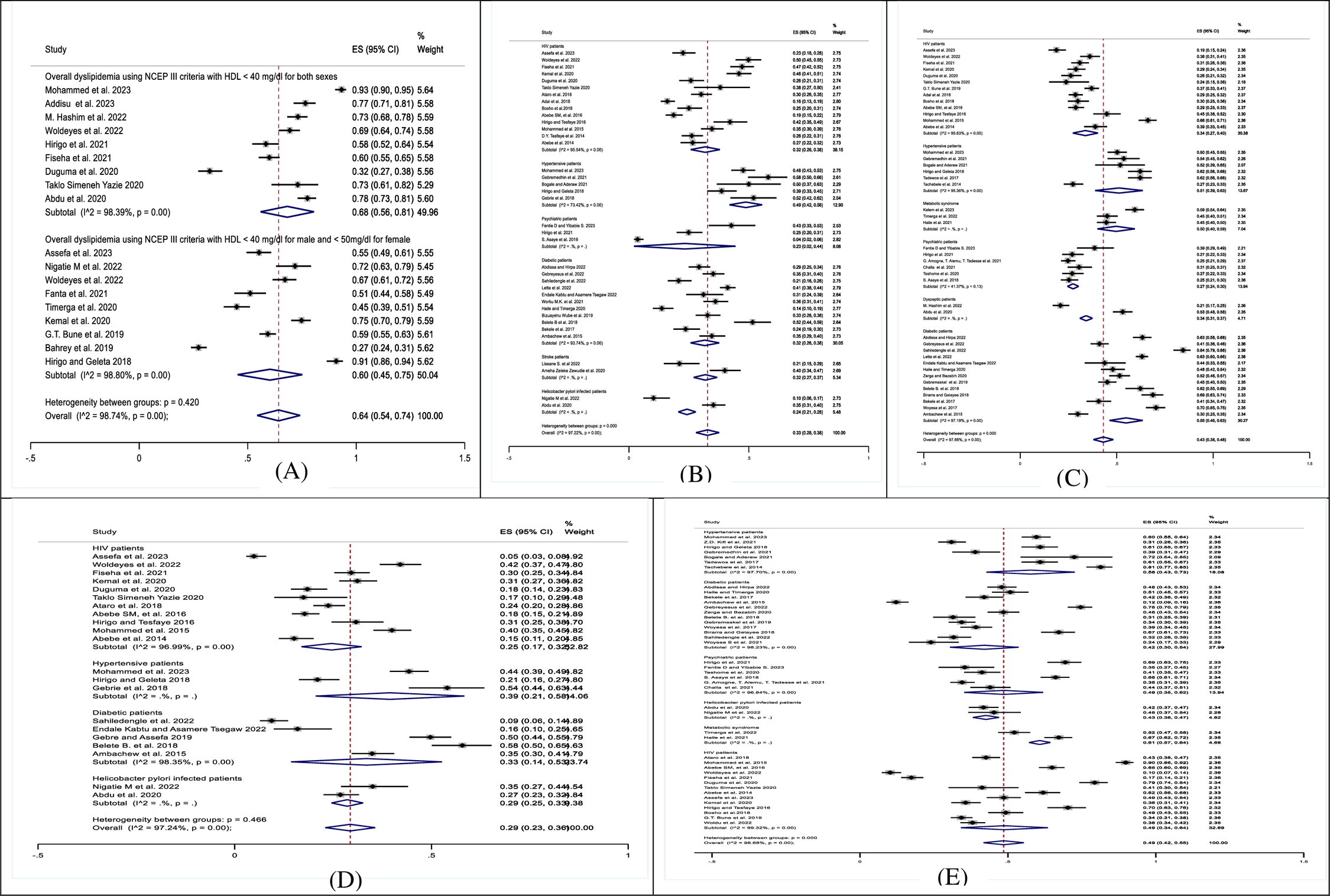

Supplement: S2 Fig — Forest plots of pooled prevalence of overall dyslipidemia and dyslipidemia components among patients in Ethiopia by sub-groups. A, Forest plot displaying the pooled prevalence of overall dyslipidemia by HDL-c cut-off. B, Forest plot displaying the pooled prevalence of TC by study population. C, Forest plot displaying the pooled prevalence of TG by study population. D, Forest plot displaying the pooled prevalence of LDL-c by study population. E, Forest plot displaying the pooled prevalence of HDL-c by study population. (TIF) [file pone.0320119.s005.tif]

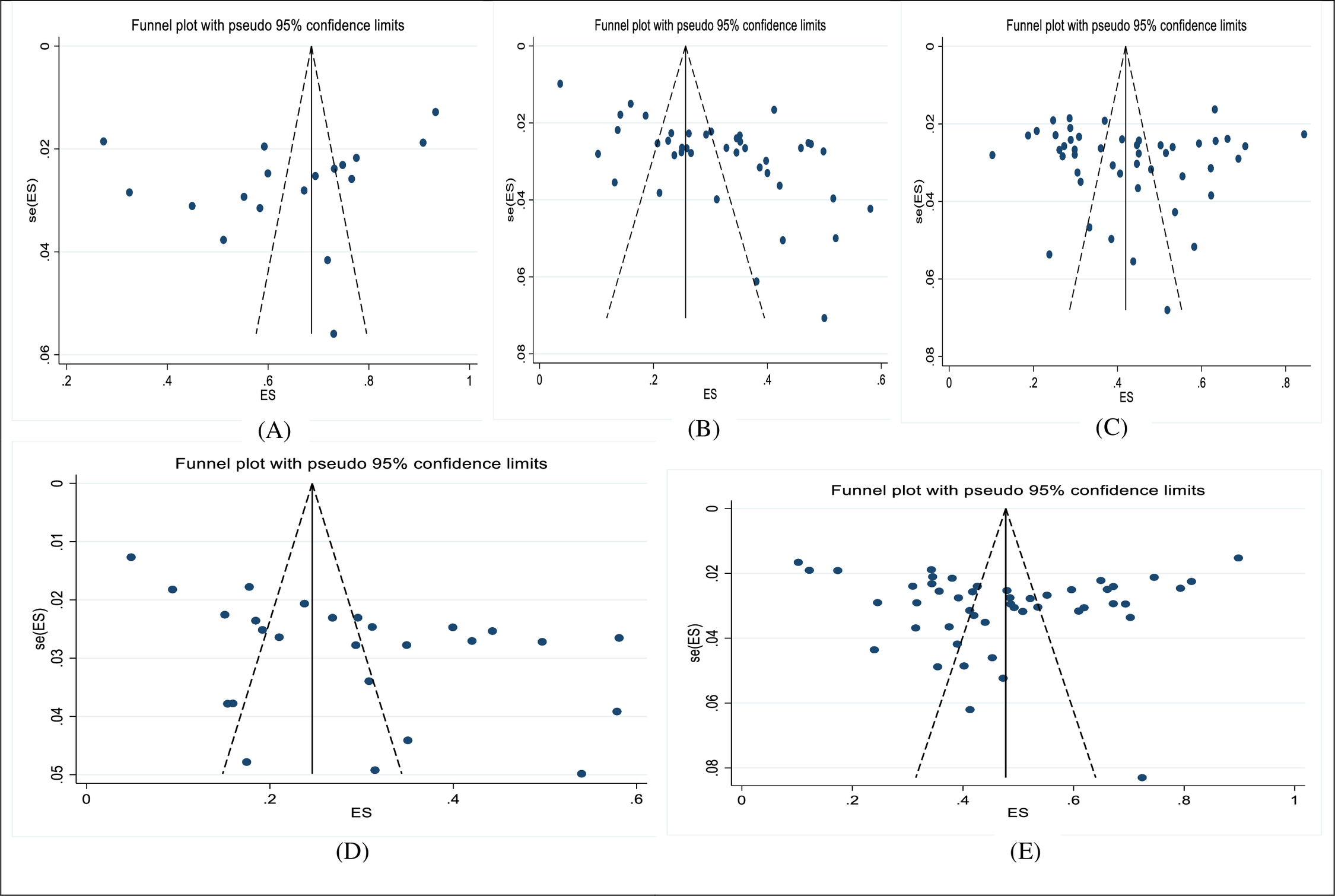

Supplement: S3 Fig — Small study effect assessed by using funnel plot for overall dyslipidemia and dyslipidemia components among patients in Ethiopia, 2023. A, Small study effect assessed by using funnel plot for overall dyslipidemia. B, Small study effect assessed by using funnel plot for TC. C, Small study effect assessed by using funnel plot for TG. D, Small study effect assessed by using funnel plot for LDL-c. E, Small study effect assessed by using funnel plot for HDL-c. (TIF) [file pone.0320119.s006.tif]

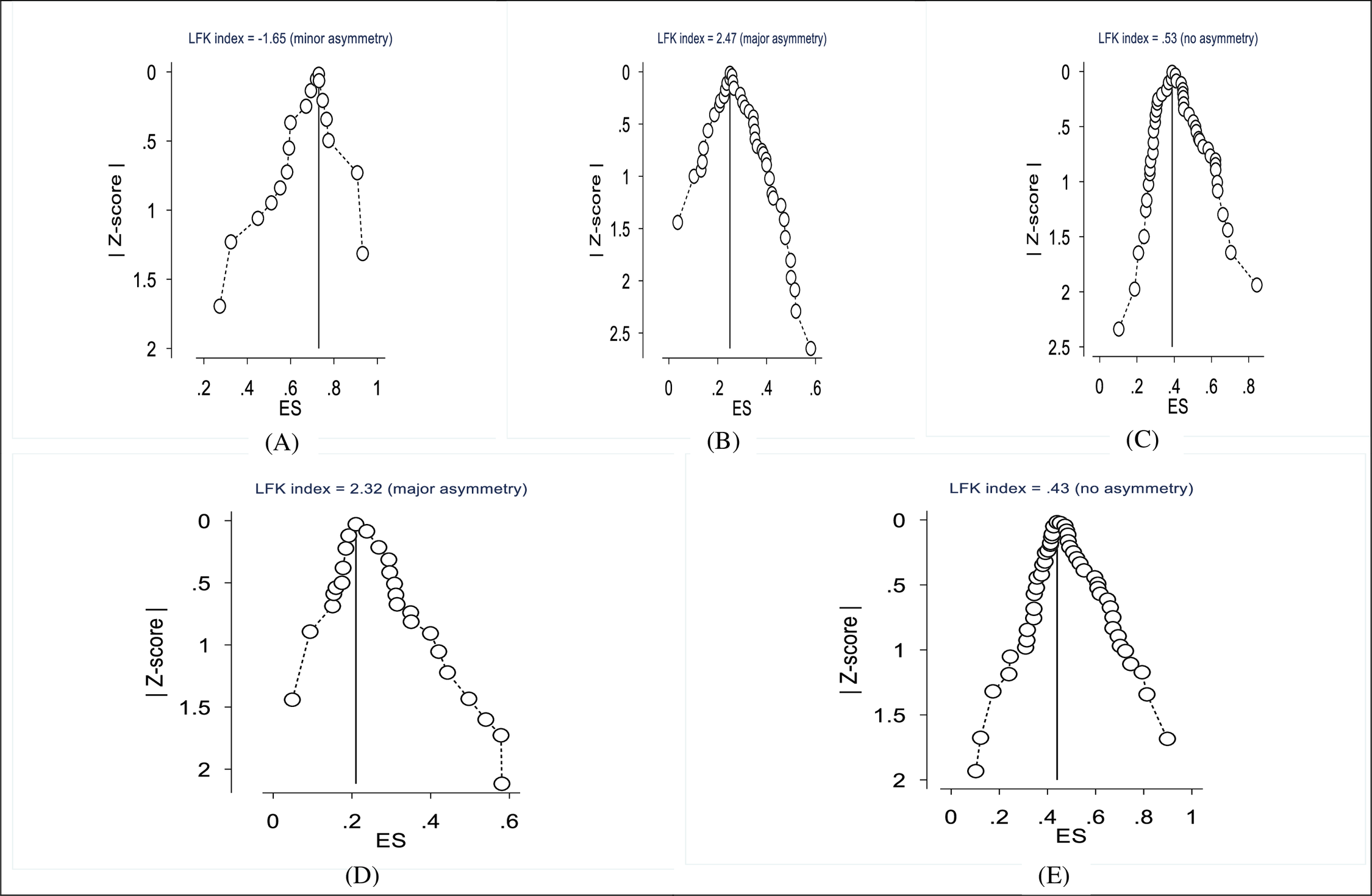

Supplement: S4 Fig — Small study effect assessed by using the LFK index for overall dyslipidemia and dyslipidemia components among patients in Ethiopia, 2023. A, Small study effect assessed by using the LFK index for overall dyslipidemia. B, Small study effect assessed by using the LFK index for TC. C, Small study effect assessed by using the LFK index for TG. D, Small study effect assessed by using the LFK index for LDL-c.E, Small study effect assessed by using the LFK index for HDL-c. (TIF) [file pone.0320119.s007.tif]
